# HER-2/*neu* Testing and Therapy in Gastroesophageal Adenocarcinoma

**DOI:** 10.4061/2011/674182

**Published:** 2010-12-06

**Authors:** Cathy B. Moelans, Paul J. van Diest, Anya N. A. Milne, G. Johan A. Offerhaus

**Affiliations:** Department of Pathology, University Medical Center Utrecht, Heidelberglaan 100, P.O. Box 85500, 3508 GA Utrecht, The Netherlands

## Abstract

Despite ongoing advances in the treatment of gastroesophageal cancer, prognosis remains poor. The best promise to improve this poor survival is provided by new targeted agents. Of these, human epidermal growth factor receptor 2 (HER2) is currently in the spotlight. In this review, we provide an overview of recent developments in HER2 testing and results of clinical trials targeting HER2 in gastroesophageal adenocarcinoma. Based on the encouraging ToGA trial findings it is now expected that routine HER2 testing will be included in the diagnostic work-up of patients with advanced gastric cancer. With regard to this testing, overexpression of the HER2 protein seems to possess the best predictive properties. However, HER2 immunohistochemistry (IHC) is subject to assay and interobserver variability, so standardization and internal and external proficiency testing is an absolute prerequisite, especially as the IHC scoring system in gastric cancer is different from that of breast cancer. Further study is needed to investigate the clinical meaning of the significant heterogeneity observed in both gene amplification and protein overexpression in gastroesophageal cancer. Highly effective therapies for gastroesophageal cancer can only be accomplished by a multi-targeted approach, considering crosstalk between pathways and continuing to optimize chemotherapy.

## 1. Introduction

Despite ongoing advances in the treatment of gastroesophageal cancer, prognosis remains poor. Esophageal adenocarcinoma incidence has been rapidly increasing in Western countries during the past half century, especially in Caucasian males. This is believed to be attributable to the increased prevalence of gastroesophageal reflux disease and its major determinant, obesity [1], resulting in Barrett's esophagus. Esophageal adenocarcinoma arising in Barrett's esophagus has a poor prognosis with a 5-year relative survival of 10–20%. Gastric cancer affects about one million people per year and is the second leading cause of cancer-related mortality worldwide [2]. Gastric cancer is thought to result from a combination of environmental factors and accumulation of specific genetic alterations, and consequently, mainly affects older patients. Gastric cancer exists as two main histological types, diffuse and intestinal, as described by Lauren [3], and can be subdivided into proximal (cardia) and distal (corpus and pylorus) cancers. Interestingly, there seems to be a trend towards more proximally (cardiac) located gastric cancer. This distal to proximal shift is yet incompletely understood and seems to parallel the observed recent rise in incidence of Barrett's esophagus. This fall in incidence in mid- and distal gastric cancer may be explained by the decline in *Helicobacter pylori *infection and associated atrophic gastritis. When a tumor is located at the gastroesophageal junction, it is often unknown whether the tumor is of esophageal or gastric origin. This group of cancers is therefore called “gastroesophageal junction cancers.” Surgery is the mainstay of treatment for resectable adenocarcinomas of stomach and esophagus, but recurrence rates are high even after radical resection. In Western countries, most patients are diagnosed at an advanced (unresectable) stage and, despite benefits of palliative radiotherapy and chemotherapy, survival of patients with advanced tumors remains poor (median survival 7–10 months) [4]. The best promise to improve this poor survival is provided by new agents acting against specific molecular targets. Of these, HER2 is currently in the spotlight. The aim of this paper is to provide an overview of recent developments in HER2 testing and results of clinical trials targeting HER2 in gastric and esophageal adenocarcinoma. Since most studies combine Barrett's esophagus, gastroesophageal junction cancers, and gastric cancer, the term “gastroesophageal” is used to refer to this combined group.

## 2. Human Epidermal Growth Factor Receptor 2

HER-2/*neu* (HER2) is a proto-oncogene located on chromosome 17q21 and a member of the human epidermal growth factor receptor (EGFR) family. It encodes a 185 kD transmembrane tyrosine kinase receptor protein that, through dimerisation with other family members, regulates signal transduction in cellular processes including proliferation, differentiation, and cellular survival [5, 6]. Many studies have indicated a role of HER2 in the development of various types of human cancer. HER2 is amplified, and the expression of its receptor protein is increased in about 10–20% of breast carcinomas [7–11]. HER2 amplification and/or overexpression have also been observed in colon [12], bladder [13], ovarian [14], Fallopian tube [15], endometrial [16], lung [17], uterine cervix [18], head and neck [19], prostate [20], pancreatic [21], salivary gland [22], and esophageal [23] and gastric [24] carcinomas.

Patients with HER2-positive (amplification and/or overexpression) primary and metastatic breast tumors have increased survival rates when treated with trastuzumab (Herceptin), a recombinant humanized monoclonal anti-HER2 antibody [25, 26]. The efficacy of trastuzumab in breast cancer patients urged investigation into its antitumor activity in patients with other HER2-positive cancers, including gastroesophageal cancer. Furthermore, overexpression [27] and amplification of HER2 [28] have also been shown to correlate with poor prognosis and with resistance to conventional adjuvant chemotherapy and tamoxifen [29–33] in breast cancer. With the recognition of its prognostic, predictive, and therapeutic implications, assessment of HER2 status has now become of major importance in clinical practice for cancer patients.

### 2.1. Diagnostic Tests to Detect HER2 Amplification and Overexpression

Since the costs for trastuzumab therapy are high and side effects are significant, accurate selection of eligible patients for this therapy is crucial. Since 1998, trastuzumab has been used to treat more than 740,000 patients with HER2-positive breast cancer worldwide, so there is much to learn from the diagnostic methods used in the selection of breast cancer patients for this treatment. HER2 status is mainly assessed by immunohistochemistry (IHC) and chromogenic (CISH) or fluorescence *in situ* hybridization (FISH). 

At present, the most common method to assess HER2 status is IHC [11] which is a routine technique available in most pathology laboratories to detect protein expression levels. Among HER2 IHC scoring systems for breast cancer, the HercepTest (Dako, Glostrup, Denmark) is frequently used to evaluate patterns of membranous immunoreactivity on tumor cells. The scoring system is based on intensity of reactivity, whether complete or incomplete and the percentage of reactive cells. Patterns are scored as IHC 0 (no staining or staining in <10% of tumor cells, negative), IHC 1+ (faint/barely perceptible incomplete membrane staining in >10% of tumor cells, negative), IHC 2+ (weak to moderate complete membrane staining in >10% of tumor cells, equivocal), or IHC 3+ (strong complete membrane staining in >10% (until 2007) or >30% (2007–now) of tumor cells, positive) [34]. Although staining and scoring methodology has been better standardized with the introduction of the Hercep Test than for most IHC assays, IHC is affected by poor tissue fixation, and there are still problems with reproducibility and interpretation of IHC assays [35–37], leading to both false negative and false positive IHC results. In addition, in breast cancer, there is some evidence that testing for HER2 gene amplification provides better predictive information than IHC [38–41]. Gene amplification testing was traditionally mostly done by FISH. For FISH assessments, an HER2:CEP17 (centromeric probe 17) ratio of >2.2 is now used (≥2.0 before 2007) to define HER2 positivity (amplification), and ratios of 1.8–2.2 and <1.8 are used to define equivocal and negative categories, respectively [34].

Comparative studies of FISH and IHC have generally shown a high level of concordance in breast cancer [39, 42, 43]. Discordant results were mainly observed for tumors that were scored 2+ by IHC. However, pathologists have been reluctant to embrace routine FISH testing, because it is a difficult, expensive, and cumbersome technique that requires trained personnel which is not available in every pathology laboratory. Moreover, fluorescence fades upon storage, making it difficult to preserve the slides for future reference, and the fluorescent probes in the kits have a limited half life. Furthermore, detailed morphological features of the tumor are usually difficult to observe due to the required protein digestion and the fluorescent mode, and heterogeneity can be missed since spots are evaluated at ×100 magnification using oil immersion. 

Chromogenic *in situ* hybridization (CISH) was introduced as an alternative for FISH in 2000 by Tanner et al. [44], using an immunoperoxidase reaction to detect specific DNA probes, which makes visualization possible with a conventional bright field microscope. Furthermore, similar to IHC, a permanent staining record is retained, and better morphologic examination is possible facilitating detection of heterogeneity. This is important in gastroesophageal cancer since higher rates of heterogeneity have been reported in gastric cancer (5%) compared to breast cancer (1.5%) [45]. CISH is also easier to interpret for pathologists who are not trained in fluorescence microscopy, and it is less expensive than FISH. In CISH scoring, the presence of large peroxidase-positive intranuclear clusters or >10 individual small signals in >50% of tumor cells (counted in at least 20 tumor cells) indicates HER2-positivity (amplification). The presence of small peroxidase-positive intranuclear clusters or 6–10 individual small signals indicates a low-level amplification, and 5 or less individual small signals are scored as HER2-negative [34]. 

In several breast cancer studies, HER2 CISH correlated well with FISH and IHC [44, 46–51]. In gastric cancer, one study systematically analyzed the concordance between CISH and FISH assays and observed a perfect correlation in 128 samples [52]. However, one drawback of CISH assays is that amplification can only be assessed semiquantitatively. Therefore, detection of amplification by easier quantitative PCR techniques has been proposed as an alternative. One of the newly introduced techniques for detection of HER2 amplification in breast cancer is multiplex ligation-dependent probe amplification (MLPA) [53]. This technique determines relative copy numbers in a quantitative way and requires only minute quantities of small DNA fragments, which makes it very suitable for DNA isolated from paraffin-embedded material. In previous studies in breast cancer, excellent results were obtained with MLPA in comparison with IHC, CISH and FISH [10, 11, 51, 54, 55]. All currently available Food and Drug Administration approved or Clinical Laboratory Improvement Amendment validated HER2 tests have been recently summarized by Allison [56].

To eliminate discrepancies observed between IHC and FISH, Hofmann et al. [45] established an IHC scoring system specific for gastric cancer. In an international consensus meeting, modifications to the breast scoring system were made mainly based on the more frequent basolateral (incomplete) membrane staining and heterogeneity in gastric cancer. This new scoring system, illustrated in Table 1, has also been used to select patients for a clinical trial to evaluate trastuzumab efficacy and safety in HER2-positive advanced esophageal and gastric cancer [57]. A subsequent study validated these guidelines in terms of interlaboratory and interobserver consensus in a large series of gastric cancer and formulated additional specific recommendations [58]. For example, for reproducible intensity scoring, the degree of microscopic magnification (×-fold) at which membranous (linear intercellular) staining is clearly visible should be considered. Strong tumor HER2 IHC staining is usually already directly visible. In these cases, only low magnification (×2.5–5) is needed to confirm strong staining intensity. In any case where high magnification (×40) is required for unequivocal demonstration of membranous staining, the tumor is scored IHC 1+. The interobserver variation results within a ring-study prior and after application of the magnification rule clearly were in favor of such an approach over nonstandardized wording, for example, of “barely visible” for IHC 1+.

### 2.2. Relationship between HER2 Amplification and HER2 Overexpression

Nine studies (totalling 1,232 samples) examining the frequency of *HER2* amplification in gastroesophageal cancer showed a mean *HER2* positivity rate of 19.2% (range 7–43%) [45], which is similar to the reported percentage of protein overexpression. 

**Figure 1:**
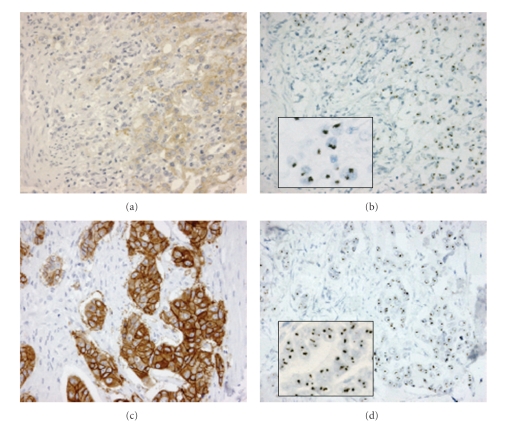
Two gastric tumors analyzed by HER2 immunohistochemistry (IHC, HercepTest) and chromogenic *in situ* hybridization (CISH). (a) Case 1 with IHC 2+ score and corresponding (b) CISH amplification (see inset). (c) Case 2 with IHC 3+ score and corresponding (d) CISH amplification (see inset).

In breast cancer, it is generally thought that HER2 overexpression is the direct result of gene amplification [59]. In esophageal and gastric cancer, concordance percentages between amplification and overexpression reported in literature range between 86.9 and 96.4% [60], as illustrated in Figure 1. Nevertheless, primary results from a very recent phase III trial (ToGA) containing >3,800 advanced esophageal and gastric cancer samples showed that the frequency of samples with amplification but without corresponding overexpression was high (23%) compared to that in breast cancer suggesting that FISH testing may be the more relevant procedure to conduct on these tumor specimens [61]. However, preliminary data from this same trial reported that patients with amplified tumors without overexpression (IHC 0 or 1+) did not show a substantial overall survival benefit from trastuzumab (HR 1.07, median overall survival 10.0 months versus 8.7 months) in contrast to patients with IHC 2+/FISH positive or IHC 3+ tumors (HR 0.65, median overall survival 16.0 months versus 11.8 months) [57], suggesting that measuring HER2 at the protein level should be the primary screening method for selecting gastroesophageal cancer patients for trastuzumab therapy. Final publication of these trial data needs to be awaited to draw firmer conclusions, and further research may be necessary to clarify these findings. The pattern seen in breast cancer, where amplification of HER2 leads to an overexpression of the protein, does not seem to have been fully confirmed in gastric cancer yet. If the observations in the ToGA trial are correct, this might be similar to what has been reported with another gene/protein relationship in breast cancer: topoisomerase II alpha (TOP2A). Unlike TOP2A, HER2 protein expression is not cell-cycle dependent, so other mechanisms (increased receptor degradation, transcriptional repression) may lay behind this discrepancy. Also, gastric cancer may have more inherent genomic instability than breast cancer resulting in more genes which are amplified as a bystander effect and not necessarily resulting in a functional increase of protein expression.

### 2.3. HER2 Expression in Gastroesophageal Cancer

Reported rates of HER2 overexpression in gastroesophageal cancer vary widely (2–45%) due to small sample sizes, differences in patient populations and methodological and scoring differences between studies [45, 62–67]. In addition, differences between HER2 overexpression in European and Asian/South-American populations (22–28% versus 3–15%, resp.) have been reported by some studies [68], but others have found these differences to be less substantial [69]. The largest data set of >3,800 advanced esophageal and gastric cancer samples found HER2 protein positivity rates of 23% [57, 68]. A recent review combining data from 24 studies (6,542 patients) calculated a weighted mean of 19% HER2 positivity [60]. In the few studies that reported separate HER2 positivity rates for gastroesophageal junction cancers and gastric cancer, HER2 positivity was higher in gastroesophageal junction cancer (24–35%) than in gastric cancer (9.5–21%) [65, 68, 70, 71].

### 2.4. HER2 in Esophageal Cancer

The data on HER2 overexpression in esophageal cancer are variable, with most studies showing HER2 overexpression in 9%–60% of cases, whereas other reports failed to observe HER2 expression [72]. The differences among reported overexpression rates might depend on stage of the disease, tumor histology (adenocarcinoma versus squamous cell carcinoma), methodology, and interpretation of IHC results. The relationship between HER2 expression and the prognosis of patients with esophageal cancer is not clear. It has been demonstrated that HER2 overexpression correlates with tumor invasion and lymph node metastasis, and thus indicates a poor prognosis [71, 73–75]. 

Studies that specifically analyzed HER2 expression and/or amplification in Barrett's esophagus reported positivity rates of 38–50% and showed an association with progression from Barrett's esophagus to dysplasia and adenocarcinoma [76–79]. A very small pilot study showed that trastuzumab treatment caused HER2 downregulation and increased apoptosis in patients with dysplasia and adenocarcinoma arising in Barrett's esophagus [80].

### 2.5. HER2 in Gastric Cancer

Previous studies have shown that early-onset gastric cancer (presenting at the age of 45 years or younger) forms a small (<10%) [81] but distinct group of gastric cancers with a different molecular expression profile than conventional gastric cancer [82–84]. We recently showed that these younger patients show very low (<5%) HER2 amplification and overexpression frequencies (unpublished data). Common gastric tumors classified as intestinal type are more likely to be HER2-positive (16–34%) than diffuse (2–7%) or mixed (5–20%) types [65, 68, 70]. The reason for the selective overexpression of HER2 in intestinal-type gastric cancers is complex and needs further investigation. The association of HER2 with a specific type suggests that intestinal- and diffuse-type gastric cancers develop along different molecular pathways and supports earlier studies showing distinct patterns of genetic alterations in gastric cancers of differing histopathologic features [85]. Some similarities can be drawn with breast cancer: diffuse-type gastric cancers and lobular invasive breast carcinomas are both associated with E-cadherin loss, which is inversely correlated with HER2 amplification/overexpression which is more common in ductal invasive breast carcinomas and intestinal-type gastric cancers.

Although some studies have reported that HER2 amplification and overexpression are highly homogeneous within a tumor and between primary and metastatic gastric cancer [62], others have reported significant heterogeneity in both gene amplification and protein overexpression in individual cancers, even among IHC 3+ cancers [67, 86]. Some studies, including our own findings (unpublished data), showed homogeneous *HER2* gene amplification but heterogeneous HER2 protein expression in certain samples, indicating that false negatives might arise when IHC is employed to predict trastuzumab response, especially when insufficient material is examined, such as in gastric biopsy specimens [52]. Generally, higher rates of HER2 heterogeneity have been reported in gastric cancer (5%) compared to breast cancer (1.5%) [45]. Chromosomal instability is probably one of the major causes of this heterogeneity. It needs to be shown whether patients with small cohesive HER2-positive clones show a different response to trastuzumab compared with patients with extended HER2-positive areas.

Although reports are conflicting, some studies have suggested that HER2-positive status in gastric cancer is associated with poor outcomes and aggressive disease [65, 70].

## 3. Preclinical and Clinical Data: Anti-HER2 Therapy

Several studies have indicated antitumor activity of trastuzumab and lapatinib in human gastric cancer cell lines (NCI-N87, 4-1ST, SMU-216, MKN-45P) or xenograft models which overexpress HER2 [65, 87–90]. In these preclinical studies, these targeted compounds have been shown to be effective both as single agents and in combination with chemotherapeutic agents that are widely used for the treatment of gastric cancer. The three-drug combination of capecitabine (Xeloda, Roche), cisplatin, and trastuzumab showed a remarkable tumor inhibitory effect in the NCI-N87 tumor xenograft model, and it is this drug combination that was also used in the ToGA trial. This trial was the first randomized, prospective, multicenter, phase III trial to study the efficacy and safety of first-line trastuzumab in HER2-positive advanced gastroesophageal cancer [60, 68]. The modest but clinically significant overall survival benefit indicated that trastuzumab is a new, effective, and well tolerated treatment for HER2-positive gastroesophageal cancer. In this trial, patients with gastroesophageal cancer (*n* = 3,807) were centrally tested for HER2 status by IHC and FISH (patients were eligible if their tumor samples were scored as 3+ on IHC or if they were FISH positive (HER2:CEP17 ratio ≥ 2.0), and 22% were HER2 positive. HER2 positivity was higher in esophageal junction cancers (33%) than gastric cancer (21%), and tumors classified as intestinal type (32%) were significantly more likely to be HER2-positive than diffuse (only 6%) or mixed (20%) types. Median overall survival, the primary endpoint, was significantly prolonged in the trastuzumab plus chemotherapy arm when compared with chemotherapy alone (13.5 months versus 11.1 months; *P* = .0048), representing a 26% reduction in the risk of death in the trastuzumab group (hazard ratio 0.74, confidence interval 0.60–0.91). The overall response rate was also significantly greater in the trastuzumab arm (47.3% versus 34.5%; *P* = .0017). Safety profiles were similar in the two study groups with no unexpected adverse events being reported with the addition of trastuzumab to chemotherapy.

No clinical data with lapatinib are available so far, but several phase II studies are ongoing and even a phase III trial has been initiated. Compared to trastuzumab, lapatinib is a small molecule tyrosine kinase inhibitor (TKI) that targets both HER2 and epidermal growth factor receptor (EGFR/HER1) and can be administered orally. It would be interesting to see whether the dual action of lapatinib will provide additional benefit over trastuzumab, especially in view of the fact that EGFR also seems to be overexpressed in gastric cancer [91–93]. 

## 4. Changing Treatment of Esophageal/Gastric Cancer

Despite advances in clinical diagnostics, surgical techniques, chemotherapy, and radiotherapy regimens, prognosis of gastric cancer remains poor, and novel treatment options as well as predictors of treatment response are urgently needed. Van Cutsem et al. presented preliminary results of the ToGA study at the 2009 ASCO Annual Meeting [94]. Although data from this trial should be considered encouraging and a major step forward in the treatment of advanced gastric cancer, some important considerations should be made. Firstly, the absolute benefit in response to trastuzumab addition to chemotherapy was 12.8%, indicating that—as in breast cancer—there is also resistance to trastuzumab even among HER2-positive selected patients. A better understanding of HER but also of other signalling pathways such as the Wnt and TGFb pathways is therefore crucial. A combination of targeted agents, which ideally target different “crosstalk” pathways, would theoretically lead to highly effective therapies [95]. Secondly, although trastuzumab efficacy is likely in the adjuvant setting, trastuzumab use in early gastric cancer requires the completion of new adjuvant phase III trials. Thirdly, although some studies have reported that HER2 amplification and overexpression are highly homogeneous within tumors and between primary and metastatic gastric cancer [62], others have reported significant heterogeneity in both gene amplification and protein overexpression in individual cancers, even among IHC 3+ cancers [67, 86]. This could impede predicting treatment response and thus selecting the right patients for treatment.

Future directions of research in HER2-positive gastroesophageal cancer should focus on the evaluation of novel antibodies (such as pertuzumab, a dimerization inhibitor, and T-DM1, a drug that combines trastuzumab with a linked chemotherapy agent called maytansine), irreversible tyrosine kinase inhibitors (such as neratinib and BIBW 2992, both dual EGFR-HER2 inhibitors), and inhibitors of HER2-related downstream signaling (such as mammalian target of rapamycin (mTOR), heat shock protein 90 (Hsp90), and PI3K/Akt) and of receptor crosstalk (such as other HER family members, vascular endothelial growth factor receptor (VEGFR), and insulin-like growth factor receptor (IGFR)). 

In the latter category, some promising targeted agents have already been investigated in gastroesophageal cancer. Molecular interactions between HER2 and other members of its family (HER1 or EGFR, HER3 and HER4) have led to the development of new targeted therapies such as the anti-EGFR monoclonal antibody cetuximab, the anti-EGFR oral small molecule tyrosine kinase inhibitors erlotinib and gefitinib, and the dual EGFR-HER2 tyrosine kinase inhibitor lapatinib [96]. Cetuximab has undergone more extensive evaluation in gastroesophageal cancers than any other targeted agent in the locally advanced setting as well as in the first-line metastatic setting, the second-line setting, and beyond. Unfortunately, most of these trial results have only been published in abstract form, and final publication is eagerly awaited. Cetuximab shows promise in the treatment of esophageal and gastric cancers in the locally advanced as well as in the first-line metastatic setting. Both erlotinib and gefitinib have very little single-agent activity in the first- and second-line settings in gastroesophageal cancers. Lapatinib has shown only modest results in the very few studies evaluating its activity in the metastatic setting. However, despite these modest results, the phase III LOGIC trial is currently evaluating the combination of capecitabine/oxaliplatin with or without lapatinib as first-line therapy in HER2-positive locally advanced, unresectable or metastatic gastroesophageal cancer.

HER2 has also been shown to communicate with VEGFR, and studies in breast cancer have shown a synergistic interaction with the combination of trastuzumab and a VEGFR tyrosine kinase inhibitor [97]. Therapies directed against VEGF(R) are the focus of ongoing research in many malignancies including gastroesophageal cancer [98, 99]. Bevacizumab, a monoclonal anti-VEGF antibody, has been investigated in the locally advanced and metastatic first- and second-line setting with encouraging phase II results [96]. A confirmatory international phase III trial of capecitabine/cisplatin with or without bevacizumab in advanced gastric cancer is currently underway. Sunitinib and sorafinib, both multitarget (among which VEGFR) tyrosine kinase inhibitors, have been tested in metastatic gastroesophageal cancers with promising preliminary results [96].

## 5. Conclusion and Future Directions

Based on the encouraging ToGA trial findings, it is now expected that routine HER2 testing will be included in the diagnostic work-up of patients with advanced gastric cancer. With regard to this testing, overexpression of the HER2 protein seems to possess the best predictive value. However, HER2 IHC is subject to assay and interobserver variability; so standardization and internal and external proficiency testing is an absolute prerequisite, especially because the IHC scoring system in gastric cancer is different from that of breast cancer.

As in breast cancer, trastuzumab research will now most probably move into the adjuvant setting. The combined treatment of chemotherapy and trastuzumab may also be beneficial in decreasing the recurrence of the disease after resection of the tumor.

Given the high degree of primary and acquired resistance to trastuzumab therapy and bearing in mind that most patients with gastroesophageal cancer are HER2-negative, highly effective therapies can only be accomplished by a multitargeted approach, considering crosstalk between pathways and continuing to optimize chemotherapy. 

Targeted agents will likely gain an increasingly important role in the treatment of patients with esophageal and gastric cancer in the near future, but how big a part they will play is unclear at this point. Lessons learnt over the past decades in breast cancer can help us maximize therapy benefit.

## Figures and Tables

**Table 1:** Consensus panel recommendations on HER2 scoring for gastric cancer [45, 58].

Reactivity characteristics	Score/classification
No reactivity or membranous reactivity in <10% of tumor cells	0/negative
Faint/ barely perceptible membranous reactivity in >10% of tumor cells; cells are reactive only in part of their membrane, in any case where high magnification (×40) is required for unequivocal demonstration of membranous staining	1+/negative
Weak to moderate complete or basolateral membranous reactivity in >10% of tumor cells	2+/equivocal
Moderate to strong complete or basolateral membranous reactivity in >10% of tumor cells; only low magnification (×2.5–5) is needed to confirm strong staining intensity.	3+/positive
Biopsy (not surgery) samples with cohesive either IHC3+ and/or FISH+ clones (at least 5 cells) are considered positive irrespective of size, that is <10% of tumor area	3+/positive

FISH: fluorescence *in situ* hybridization; HER2: human epidermal growth factor receptor 2; IHC: immunohistochemistry.
